# Undetected Aorto-RV Fistula With Aortic Valve Injury and Delayed Cardiac Tamponade following a Chest Stab Wound: A Case Report

**DOI:** 10.5812/traumamon.11607

**Published:** 2013-08-11

**Authors:** Jamil Esfahanizadeh, Mohammad Abbasi Tashnizi, Ali Asghar Moeinipour, Alireza Sepehri Shamloo

**Affiliations:** 1 Department of Cardiac Surgery, Imam Reza Hospital, Mashhad University of Medical Sciences, Mashhad, IR Iran; 2 Student Research Committee, School of Medicine, Mashhad University of Medical Sciences, Mashhad, IR Iran

**Keywords:** Heart Injuries, Cardiac Tamponade, Aorta, Fistula

## Abstract

**Introduction:**

Although a few patients will survive after penetrating cardiac injuries, some of them may have unnoticeable intracardiac injuries. The combination of aorto-right ventricular fistula with aortic valve injury is rare.

**Case Presentation:**

A 19 year-old man referred with an aorto-right ventricular fistula accompanied with aortic regurgitation and delayed tamponade following a stab in the chest. The patient was scheduled for fistula repair, aortic valve replacement and pericardectomy two months after trauma.

**Conclusions:**

To prevent missing intracardiac injury and also late cardiac injury complications, in all pericordial stab wounds, serial clinical examinations and serial echocardiography should be performed. In addition, cardiac injuries should be repaired during the same hospital stay.

## 1. Introduction

Penetrating cardiac injury is a serious life-threatening condition andpatients who survive, are rarely without cardiac symptoms and sings during initial evaluation. Acute cardiac tamponade due to bleeding into the pericardium is the most common clinical presentation, and almost always leads to death at the accident scene (1). On the other hand, delayed cardiac tamponade is an unusual clinical feature of heart stab wounds (2). Patients with penetrating cardiac wounds present a variety of heart and great vessels injuries which often require emergency thoracotomy and cardioraphy and about half of them survive (3). Also, such patients may require additional surgical repair for intracardiac injuries that are not diagnosed during resuscitation (4).Three to five percent of all penetrating heart injuries have concomitant intracardiac injuries involving valves, intra-arterial/ventricular septum, coronary arteries and conductive system that affect early and late outcomes of patients (5). Moreover, the intracardiac injuries make the patient`s situation more complicated. One of the rare post penetrating cardiac injuries is aorto-right ventricular fistula (Ao-RV Fistula). In one report, it comprised 0.5% of all intracardiac injuries (6). Sometimes, it may be associated with aortic valve injury and aortic regurgitation (AR) (7). King repaired the first traumatic Ao-RV fistula in 1945(8). The first post penetrating delayed cardiac tamponade was reported by MacQuot in 1920 (2, 9). So far, 19 cases of Ao- RV fistula with aortic valve injury have been reported in the English literature (7, 10-12). Herein, we report a case.

## 2. Case Presentation

**Figure 1.**
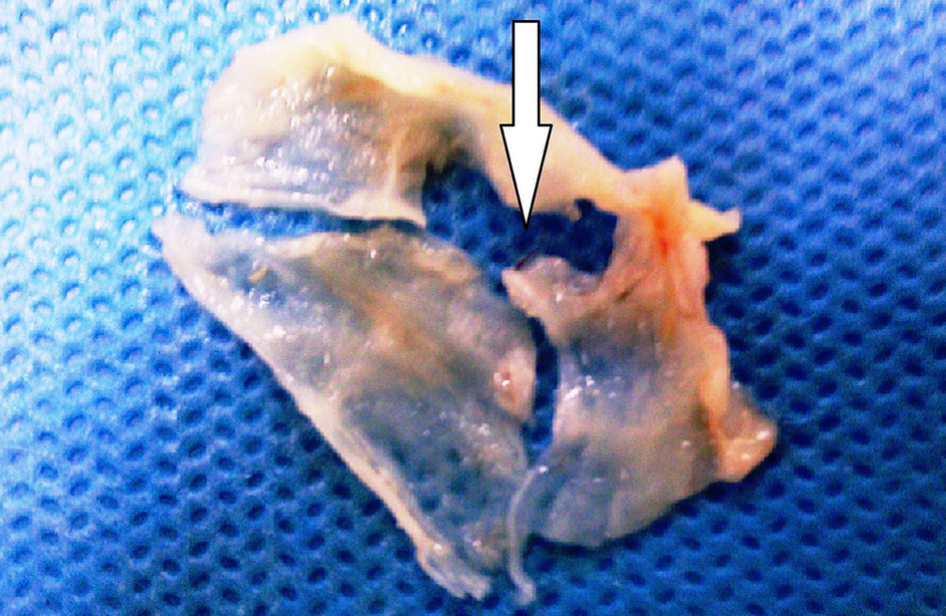
Traumatic Defect of Right Coronary Cusp (Arrow)

**Figure 2.**
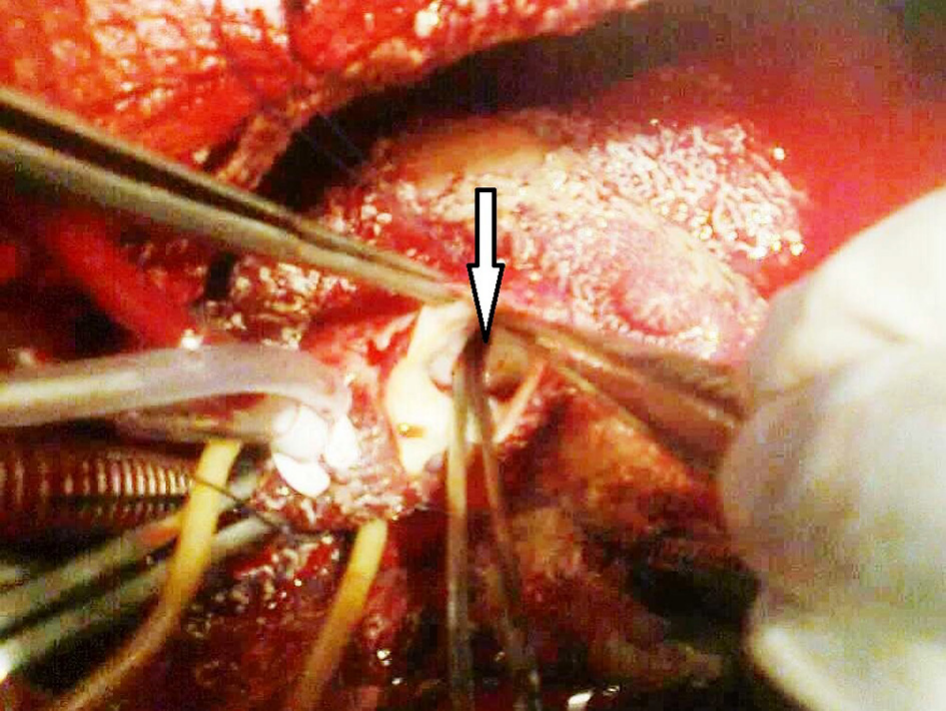
Aortic view of Aorto-RV Fistula (Arrow)

A 19-year -old healthy man, who sustained a single stab wound (1.5cm knife wound in the left 4th intercostal space para-sternally) was admitted to a local hospital. To manage hemopnumothorax, a chest tube was inserted and after 3 days the patient was discharged without any symptoms. At that time, because of no observed cardiac symptoms or signs, cardiac injury was not suspected by the general surgeon, thus, no cardiac evaluation such as echocardiography or thoracic computed tomography (CT) scan was performed. Two months later, he referred to evaluate dyspnea. He had severe shortage of breath and distending jugular veins. Lung sounds were clear but heart sounds were decreased and a continuous murmur was heard at the left sternal border (BP = 85/40mmHg, PR = 135/min, RR = 35/min, T = 38.5οC). In addition, the electrocardiogram showed sinus tachycardia, and the chest roentgenogram indicated an increased cardiac silhouette that was globular. Transthoracic echocardiography confirmed a massive pericardial effusion. Also, severe aortic regurgitation caused by right coronary cusp perforation and a defect between the right Valsalva sinus and right ventricle (RV) were demonstrated. The patient was scheduled for an urgent operation for post traumatic delayed tamponade and also intracardiac defects. After median sternotomy, a thickened and inflamed pericardium (4mm) which was full of debris and old clots was noticed. In addition, the epicardium was edematous with some adhesions. About 800cc sanguineous fluid was evacuated. Specimens of pericardial tissue and fluid were sent for culturing. Then, using aorto-bicaval cannulation, cardiopulmonary bypass and cardiac arrest were done. After aortotomy, a 10mm perforation in the right coronary cusp was found (Figure 1). Moreover; a 7mm defect in the right coronary sinus that opened into the RV was revealed (Figure 2).

A ventriculotomy was performed through the infundibulum. The defect was located 1cm below the pulmonary valve in the septum which connected to the aorta. The fistula was repaired via ventriculotomy using a Gor-Tex patch and continuous 4-0 prolene suture. Repairing the aortic valve with pericardial patch was not possible because the pericardium was inflamed. Therefore, the aortic valve was replaced by a mechanical prosthesis No.23. Finally, in order to prevent constrictive pericarditis, bilateral pericardectomy was performed. As this was an emergency operation, TEE was not available at that time the center; the patient was only evaluated with TTE postoperatively. After surgery, the patient`s hospital stay was uneventful and postoperative echocardiography demonstrated no residual shunt and pericardial effusion. The prosthetic aortic valve and left/right ventricle had good function. During 2 years of clinical follow up, the patient has been asymptomatic with normal activities.

## 3. Conclusions

Our case referred with an aorto-right ventricular fistula accompanied with aortic regurgitation and delayed tamponade following a stab in the chest. The case presented with a simple stab wound into anterior chest wall, whose cardiac injury was missed at the first admission and managed only for left hemopnumothorax. Two months later, he referred with delayed cardiac tamponade and post traumatic intracardiac defects. The patient displayed three aspects of heart injury which are unusual and rare namely showing no initial cardiac symptoms and signs, unnoticing two important intracardiac lesions (Ao-RV Fistula and aortic valve injury) and also, delayed cardiac tamponade as late sequel of penetrating cardiac trauma.

This presentation demonstrates that para-sternal penetrating trauma, even without cardiac symptoms and signs, may have cardiac and intracardiac injuries. Cardiac evaluation is necessary to exclude probable cardiac injury. Ignoring cardiac injury can lead to late cardiac complications. In the past, the incidence of post traumatic intracardiac lesions was reported to be less than 5% (5). However, thanks to echocardiography and color-flow Doppler, especially transesophageal echocardiography (TEE), these simultaneous lesions have been reported to be about 20% in recent decades (13). The most common post penetrating intracardiac lesion is ventricular septal defect (VSD) (14). Mitral and tricuspid valves injuries have been reported sporadically in traumatic patients (4, 15). Our patient had a rare heart injury with a connection between the infundibulum of the RV and right Valsalva sinus of the aorta and also aortic valve perforation created by the knife tip. Aorto-RV fistula due to injury is rare and in 35% of cases, it can be accompanied with aortic valve injury and aortic regurgitation (7). After traversing through the chest wall, a pericordial stab penetrates the anterior wall of infundibulum which can tear posterior wall of infundibulum (Conus). Then it goes through the wall of right Valsalva sinus located just behind the conus. At first, the Ao-RV fistula can be tolerated well and the patient may be asymptomatic. But, after a period of time (days or even years), it leads to heart failure and therefore, needs to be repaired. Despite the importance of physical examinations, the continuous murmur of this entity can be detected in only one-third of the patients on admission. Also, this murmur can be heard in 34% of other cases more than one week following trauma. The mean interval time to detect the murmur is reported to be 59 days after trauma (7). Therefore, a traumatic Aorto-RV fistula maybe missed at first. Furthermore, to diagnose intracardiac injuries, echocardiography provides useful information. However, in a trauma patient (emergency situation, lack of time, bleeding wounds and chest tube, pneumothorax and poor echo-window condition) initial echocardiography may not reveal cardiac damage. Consequently, it is advised to do serial echocardiography (16). Although our patient was managed only for a stab wound in the chest and hemopnumothorax, these two important intracardiac injuries (Ao-RV fistula and aortic valve injury) were missed at the first admission because he did not undergo any serial clinical and echocardiograph evaluations. Then two months later, the major patient`s clinical picture was a post traumatic delayed pericardial effusion and tamponade. It is important to consider that a missed penetrating cardiac injury may rarely lead to delayed pericardial effusion and cardiac tamponade (2, 17). A combination of post traumatic Ao-RV fistula with aortic valve perforation and delayed cardiac tamponade, similar to the current presentation, was reported by Kaya (11). Traumatic delayed pericardial effusion can cause postpericardiotomy syndrome, infectious pericarditis and secondary bleeding. Rondon et al. recommend performing echocardiography for every patient with penetrating chest injury as soon as possible after admission to the emergency department and for up to six months, because delayed pericardial effusion may occur after injury (2).

As cardiac injury can occur in every pericordial stab in the chest, serial physical examinations and serial transthoracic or even transesophageal echocardiography should be performed to exclude intracardiac injuries. In order to prevent late cardiac complications, every intrapericardial or intracardial injury ought to be evaluated and repaired at the same hospital stay.

## References

[1] Campbell NC, Thomson SR, Muckart DJ, Meumann CM, Van Middelkoop I, Botha JB (1997). Review of 1198 cases of penetrating cardiac trauma.. Br J Surg..

[2] Rendon F, Gomez Danes LH, Castro M (2004). Delayed cardiac tamponade after penetrating thoracic trauma.. Asian Cardiovasc Thorac Ann..

[3] Rodrigues AJ, Furlanetti LL, Faidiga GB, Scarpelini S, Barbosa Evora PR, de Andrade Vicente WV (2005). Penetrating cardiac injuries: a 13-year retrospective evaluation from a Brazilian trauma center.. Interact Cardiovasc Thorac Surg..

[4] Lugones I, Daneri ML, Conejeros WM, Grippo M, Schlichter AJ (2009). Acrocomia aculeata as an unreported cause of tricuspid regurgitation.. Ann Thorac Surg..

[5] Carter RL, Albert HM, Glass BA (1967). Traumatic ventricular septal defect.. Ann Thorac Surg..

[6] Beall Jr AC, Ochsner JL, Morris Jr GC, Cooley DA, Debakey ME (1961). Penetrating wounds of the heart.. J Trauma Acute Care Surg..

[7] Samuels LE, Kaufman MS, Rodriguez-Vega J, Morris RJ, Brockman SK (1998). Diagnosis and management of traumatic aorto-right ventricular fistulas.. Ann Thorac Surg..

[8] King H, Shumacker HB, Jr (1958). Surgical repair of a traumatic aortic-right ventricular fistula.. J Thorac Surg..

[9] Bellanger D, Nikas DJ, Freeman JE, Izenberg S (1996). Delayed posttraumatic tamponade.. South Med J..

[10] Hibino N, Tsuchiya K, Sasaki H, Matsumoto H, Nakajima M, Naito Y (2003). Delayed presentation of injury to the sinus of valsalva with aortic regurgitation resulting from penetrating cardiac wounds.. J Card Surg..

[11] Kaya A, Dekkers P, Loforte A, Jaarsma W, Morshuis WJ (2005). Traumatic aorto-right ventricular fistula with aortic insufficiency.. Ann Thorac Surg..

[12] Theron JP, Du Theron H, Long M, Marx JD (2009). Late presentation of aorto-right ventricular fistula and associated aortic regurgitation following penetrating chest trauma.. Cardiovasc J Afr..

[13] Skoularigis J, Essop MR, Sareli P (1994). Usefulness of transesophageal echocardiography in the early diagnosis of penetrating stab wounds to the heart.. Am J Cardiol..

[14] Antoniades L, Petrou PM, Eftychiou C, Nicolaides E (2011). A penetrating heart injury resulting in ventricular septal defect.. Hellenic J Cardiol..

[15] de Boer HD, Hamer HP, Ebels T, de Boer WJ (1998). Sharp incision of the anterior mitral leaflet due to penetrating trauma: report of a case and long-term follow-up.. Eur J Cardiothorac Surg..

[16] Jeon K, Lim WH, Kang SH, Cho I, Kim KH, Kim HK (2010). Delayed diagnosis of traumatic ventricular septal defect in penetrating chest injury: small evidence on echocardiography makes big difference.. J Cardiovasc Ultrasound..

[17] Karmy-Jones R, Yen T, Cornejo C (2002). Pericarditis after trauma resulting in delayed cardiac tamponade.. Ann Thorac Surg..

